# Colostral antibody induced interference of inactivated bluetongue serotype-8 vaccines in calves

**DOI:** 10.1186/1297-9716-42-18

**Published:** 2011-02-02

**Authors:** Damien Vitour, Jean Guillotin, Corinne Sailleau, Cyril Viarouge, Alexandra Desprat, Frédéric Wolff, Guillaume Belbis, Benoit Durand, Labib Bakkali-Kassimi, Emmanuel Breard, Stéphan Zientara, Gina Zanella

**Affiliations:** 1 UMR 1161 VIROLOGIE ANSES-INRA-ENVA, Agence nationale de sécurité sanitaire de l'alimentation, de l'environnement et du travail (ANSES), 23 avenue du Général De Gaulle, 94700 Maisons-Alfort, France; 2 Laboratoire Départemental Public, Domaine du Certia, 369 rue Jules-Guesde, BP 20039, 59651 Villeneuve-d'Ascq Cedex, France; 3 Unité Epidémiologie, Agence nationale de sécurité sanitaire de l'alimentation, de l'environnement et du travail (ANSES), 23 avenue du Général De Gaulle, 94700 Maisons-Alfort, France; 4 Pathologie du Bétail (DPASP), Ecole Nationale Vétérinaire d'Alfort (ENVA), 7 avenue du Général-De-Gaulle, 94700 Maisons-Alfort, France; 5 Groupement départemental de défense sanitaire des animaux du Nord, Maison des éleveurs, Zone d'activités, 2 Ter rue de l'Epau, 59230 Sars-et-Rosières, France

## Abstract

Since its introduction into northern Europe in 2006, bluetongue has become a major threat to animal health. While the efficacy of commercial vaccines has been clearly demonstrated in livestock, little is known regarding the effect of maternal immunity on vaccinal efficacy. Here, we have investigated the duration and amplitude of colostral antibody-induced immunity in calves born to dams vaccinated against bluetongue virus serotype 8 (BTV-8) and the extent of colostral antibody-induced interference of vaccination in these calves. Twenty-two calf-cow pairs were included in this survey. The median age at which calves became seronegative for BTV was 84 and 112 days as assayed by seroneutralisation test (SNT) and VP7 BTV competitive ELISA (cELISA), respectively. At the mean age of 118 days, 13/22 calves were immunized with inactivated BTV-8 vaccine. In most calves vaccination elicited a weak immune response, with seroconversion in only 3/13 calves. The amplitude of the humoral response to vaccination was inversely proportional to the maternal antibody level prior to vaccination. Thus, the lack of response was attributed to the persistence of virus-specific colostral antibodies that interfered with the induction of the immune response. These data suggest that the recommended age for vaccination of calves born to vaccinated dams needs to be adjusted in order to optimize vaccinal efficacy.

## Introduction

Bluetongue (BT) is a non-contagious, arthropod-borne viral disease affecting sheep, some species of wild ruminants such as deer, and to a lesser extent, cattle and goats. BT is a major concern in the international trade of animals and animal products. *Bluetongue virus *(BTV), the etiologic agent of the disease, is the type species of the genus *Orbivirus *within the family *Reoviridae*. It is transmitted almost exclusively by the bite of *Culicoides *midges. Since 1998, five distinct serotypes of BTV (1, 2, 4, 9 and 16) have spread across southern and central Europe [1,2]. In August 2006, a sixth serotype, BTV-8, was first identified in northern Europe, from where it quickly spread throughout the Netherlands, Belgium, Luxembourg, Germany and northern France [3-6]. The virus overwintered successfully in the same regions and then spread to the United Kingdom, Denmark, Switzerland and the Czech Republic in 2007. The BTV-8 strain circulating in northern Europe exhibits several unusual properties, and notably its ability to cause disease and mortality in cattle [2,7]. Mass vaccination campaigns were quickly instituted to limit the spread and the dramatic socioeconomic consequences of the BT outbreak in Europe. Commercially available inactivated vaccines are now widely used to control BTV infection, and since 2008 vaccination of cattle is compulsory in some endemic European countries. An issue that was not sufficiently addressed prior to approval of inactivated BTV vaccines was the optimum age at which calves born to vaccinated dams should be vaccinated so as to avoid colostral antibody-induced interference.

Newborn calves acquire passive immunity from their dams by ingestion and absorption of antibodies present in colostrum. The estimated duration and benefit of this passively derived humoral immunity can vary greatly depending on the colostrum production (quantity and quality) and the quantity of antibody ingested and absorbed. Maternally derived immunity can confer protection against a broad range of viral pathogens including bovine herpesvirus-1 (BHV-1), bovine viral diarrhea virus (BVDV) and bovine respiratory syncytial virus (BRSV) [8-12]. Passive immunity frequently blocks the production of serum antibodies when immunogens are administered to calves with maternally derived antibodies [13], even if in some cases immunogens can induce immunological memory that is not susceptible to maternal antibody regulation [14,15]. Also, vaccination against BHV-1 and BRSV with modified live virus (MLV) vaccines can generate immune responses such as lymphocyte blastogenesis in calves with maternal antibodies to BHV-1 and BRSV [16]. However, very few data are available as regards the duration and effect of maternally acquired immunity against BTV in calves that were born to vaccinated cows. This prompted us to investigate: (1) the time required for nursing beef calves to become seronegative; (2) the effect of colostral antibodies on the humoral response in calves after vaccination with an inactivated BTV-8 vaccine.

## Materials and methods

### Animals

Twenty-two pairs of calves-pregnant cattle originating from two distinct farms located in the north of France (Tour and Font) were included in the survey. All cows were vaccinated in May 2008 with an inactivated BTV-8 vaccine (Bovilis BTV8; Intervet) which was administered subcutaneously according to the manufacturer's instructions. The vaccinated cows were all seropositive for BTV immediately prior to calving, as determined by a VP7-specific BTV competition ELISA (cELISA) test (data not shown). The cattle presented no clinical symptoms and no BTV RNA was detected by real-time PCR (BTVM-Kit TAQVET™; LSI, France; data not shown). The calves were born in October 2008 under standard management conditions. Blood samples were collected from calves at five time points; the first sample (S1) was collected at approximately 48 days post-calving (range 36-60), S2 at 80 days (range 70-90), S3 at 111 days (range 102-122), S4 at 139 days (range 127-150) and S5 at 202 days (range 189-207). Calves were tested for the presence of BTV antibodies by cELISA and serum neutralisation test (SNT). At a mean age of 118 days, 13/22 calves (3 from Tour and 10 from Font) were vaccinated with the Bovilis BTV-8 inactivated vaccine. The vaccine was administered as 2 subcutaneous injections 3 weeks apart.

### cELISA

BTV antibody levels were measured using the cELISA ID Screen^® ^Bluetongue Competition assay (ID VET, Montpellier, France) according to the manufacturer's instructions. Results are expressed as percentage of negativity (PN) compared with the kit control and designated as positive, doubtful or negative according to the cut-off values recommended by the manufacturer (PN ≤ 35 is positive; 35 < PN ≤ 45 is doubtful; PN > 45 is negative). Statistical analyses were performed using a threshold value of 35 to discriminate between positive (PN ≤ 35) and negative (PN > 35) BTV cELISA results (see below).

### Serum Neutralisation Test (SNT)

SNT was performed using the BTV-8 South African reference strain and serotype-specific BTV-8 positive control antisera. Briefly, 50 μL of sera were diluted (1:2 to 1:256) and titrated against 100 TCID_50 _(50 μL) of the BTV-8 South African reference virus. Plates were incubated for 1 h at 37°C and then 100 μL (2 × 10^4^) of a Vero (African green monkey kidney) cell suspension was added per well. After incubation at 37°C for 5-7 days, the wells were scored for cytopathic effect (CPE). The neutralisation titre was defined as the dilution of serum resulting in 50% neutralisation endpoint. Titres were expressed as the log10 of the reciprocal endpoint serum dilution. The positive-negative cut-off was 0.9 (titre < 0.9: negative; titre ≥ 0.9: positive).

### Statistical analyses

The interval after birth when calves suckling seropositive cows became seronegative was determined using the non-parametric survival Kaplan-Meier method. Survival analysis was used to address data describing how long it takes for an event to occur. In this study, the event is the fact that an animal becomes seronegative. This event was not observed in some calves that were still seropositive prior to vaccination: these were treated as censored observations. Independent survival analyses were conducted using the cELISA and SNT results.

A Kappa value was computed to determine the agreement between cELISA and SNT in sera from non-vaccinated animals (sera from vaccinated animals collected before the vaccination date was also included). Kappa values were interpreted according to Dohoo et al. [17].

The success of BTV vaccination was defined on the basis of cELISA performed on samples collected three months after the first vaccination. Analysis of variance (ANOVA) was used to assess the existence of a relationship between vaccination success and cELISA response at S1, S2 and S3. A receiver operating characteristic (ROC) analysis [18] was then conducted to model the probability of vaccination success according to the cELISA response at S3. The cELISA cut-off resulting in optimal predictions of vaccination success was determined. In order to establish the relation between age at vaccination and vaccination success, we used the preceding cELISA cut-off to study the time interval until cELISA rises above this value (corresponding to a negative status relatively to this cut-off). The Kaplan-Meier survival analysis method was used to compute the age at which vaccination would succeed in 50% of calves.

All statistical analysis and graphs were performed with R [19].

## Results

### Correlation between SNT and ELISA

**Figure 1:**
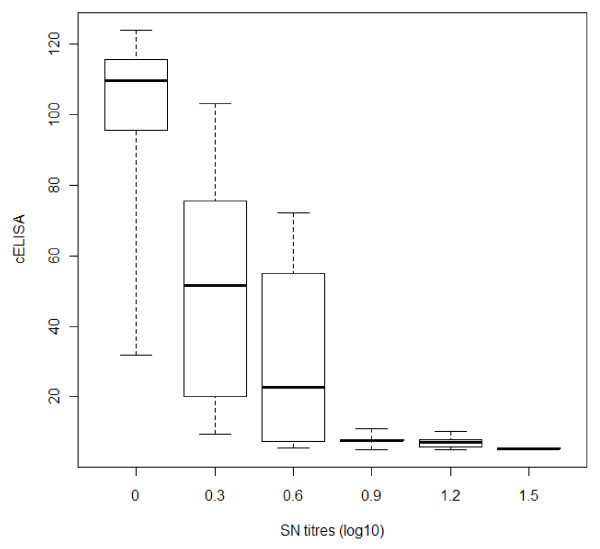
**cELISA and SNT results box-plots in non-vaccinated calves (line indicates median value, box indicates interquartile range and bars indicate range)**.

All 22 colostrum-fed calves were seropositive for BTV at the first sampling, as determined by both SNT and cELISA (i.e. S1, 36-60 day old calves). Twenty two pairs of results were used to compare cELISA and SNT agreement in calves before vaccination (Table 1); the overall kappa value (0.49; 95% confidence interval [CI]: 0.3-0.7) indicated a moderate agreement between the tests. Concordance was higher in BTV positive serums as shown in Figure 1. The result was confirmed by kappa estimates computed at each time point before vaccination. At S1 the agreement was perfect, as all animals were positive using both tests. The agreement was poor at S2 (0.07; 95% CI: -0.2-0.3) and fair at S3 (0.24; 95% CI: -0.4-0.9).

**Table 1:** BTV antibody detection in serum samples from non-vaccinated calves using cELISA and SNT.

		cELISA		
		
		Positive	Negative	Total
SNT	Positive	17	0	17
	Negative	3	2	5
	Total	20	2	22

### Estimated time to seronegative status using survival analysis

**Figure 2:**
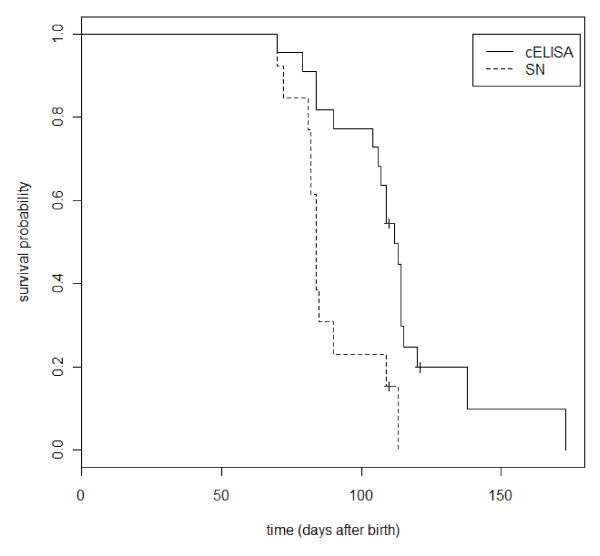
**Kaplan-Meier survival curves for time to BTV maternal antibodies seronegativation in 22 calves tested with cELISA and SN tests**.

Kaplan Meier survival curves showed that the apparent interval after birth required for loss of passively acquired antibodies depended upon the serological test used, and was found to be earlier by SNT (Figure 2). The median time after birth when calves born to seropositive cows become seronegative was 112 days by cELISA (range 70 to 173) and 84 days by SNT (range 70 to 113 days).

### Antibody response to BTV vaccination

**Figure 3:**
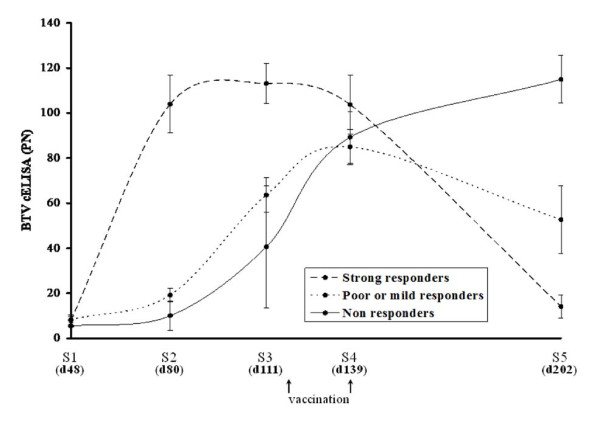
**Kinetic of BTV antibody level in vaccinated calves as measured by VP7 cELISA**. Results are expressed as mean of percentage of negativity (PN). A threshold value of 35 was defined to discriminate between positive (PN ≤ 35) and negative (PN > 45) cELISA results. Animals were sampled five times post-calving (S1 to 5). The mean age of the calves is given in days (d) in parenthesis for each sample. Calves were vaccinated at the mean age of 118 days (i.e. between S3 and 4). The group of calves that become seropositive after vaccination (strong responders) are indicated in dashed line. Mild and non responders groups are labeled using doted and plain line respectively.

Table 2 shows the cELISA PN for each vaccinated calf at each interval after initial vaccination including the serological status at S5 (202 day old calves). The data shows three different types of response following vaccination: non-responders (BTV cELISA PN higher than before vaccination), mild responders (decrease in BTV cELISA level compared with the level before vaccination but without seroconversion) and strong responders (seroconversion after vaccination) (Table 2 and Figure 3). In sample 1 (S1, mean age of 48 days), the BTV antibody level was strong for all animals regardless of group response and, therefore, ANOVA results were not significant. A statistical association was found between the response after vaccination and the cELISA levels in the three groups at S2 (mean age of 80 days) and S3 (mean age of 111 days) (p < 0.001). Indeed, at S2, while mild and non-responders remained BTV seropositive with cELISA values ranging from 5 to 23, all future responders already showed antibody levels far below the cut-off limit of the test (Table 2 and Figure 3). This trend was also confirmed immediately before vaccination, at S3, with the group of responders displaying cELISA PN > 103 whereas the antibody level in poor and non-responders was PN < 76 (Figure 3).

**Table 2:** cELISA PN antibody levels in vaccinated animals before vaccination and seroconversion status.

Population	N#	S1 (48 d)	S2 (80 d)	S3 (111 d)	S4 (139 d)	S5 (202 d)	Seroconversion status^a^
Font	1	6	5	8	70	126	Neg
	2	6	14	41	76	114	Neg
	3	7	108	119	89	15	Pos
	4	5	8	44	95	115	Neg
	5	5	7	25	92	129	Neg
	6	4	8	16	96	111	Neg
	7	5	6	76	97	115	Neg
	8	6	23	75	99	97	Neg
	9	6	114	117	113	19	Pos
	10	11	90	103	110	9	Pos
Tour	A	7	17	62	78	43	Dbt
	B	8	18	57	84	45	Neg
	C	10	23	72	93	70	Neg
Total		8 (6-11)	104 (90-114)	113 (103-119)	104 (89-113)	14 (9-19)	Pos (3/13)
		6 (5-10)	13 (5-23)	48 (8-76)	88 (70-99)	96 (43-129)	Neg or Dbt (10/13)

Strong responders		6-11	90-114	103-119	89-113	< 19	Pos (3/3)
Mild responders		7-10	17-23	57-72	78-93	43-70	Pos (0/3)
Non responders		5-6	5-24	8-76	70-99	> 96	Pos (0/7)

PN ≤ 35 is positive (pos); 35 < PN ≤ 45 is doubtful (dbt); PN > 45 is negative. The mean age of the calves is given in days (d) in parenthesis for each sample (S). Calves were vaccinated at the mean age of 118 days (i.e. between S3 and 4).

### cELISA level and vaccination success

After being shown to be inversely related to the humoral response elicited by vaccination, cELISA titres immediately prior to vaccination (S3) were used for ROC analysis. A sensitivity and specificity of 100% were obtained with cELISA cut-off values between 76 and 103.

**Figure 4:**
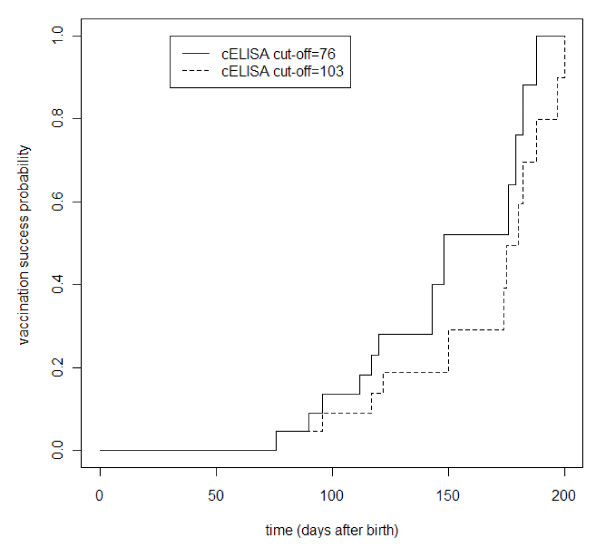
**Vaccination success probability using two cut-off cELISA values in 22 calves**.

To establish the relation between age at vaccination and vaccination success, we studied the time interval until cELISA becomes > 76 or > 103. As 6 days elapsed between the third sampling date and the date of vaccination, 6 days were added to these time intervals. The resulting time intervals were shorter with the 76 cELISA cut-off value than with the 103 cut-off value, as shown in Figure 4. Median time for the 76 cut-off was 148 days (range 76 to 188 days). For the 103 cut-off value median time was 180 days (range 76 to 197 days). These results indicate that in this animal sample vaccination would have succeeded in 50% of the animals, if they had been vaccinated between 148 and 180 days of age.

## Discussion

Following the 2006 outbreak of BTV-8 in livestock in northern Europe, BT has become a major concern in animal health and trade. Vaccine manufacturers rapidly provided efficacious and safe inactivated BTV-8 vaccines that are now widely and successfully used throughout Europe to control BTV infection [20,21]. However, extensive studies have not been performed to evaluate the effects of passive immunity in calves born to immunized dams on the humoral response to BTV-8 immunization. In this survey, we followed the kinetics of loss of BTV-8 specific colostral antibodies in calves born to vaccinated dams using SNT and cELISA assays. Comparison of the antibody levels observed in these two tests showed only a moderate agreement, likely reflecting the fact that cELISA and SNT measure distinct antibody populations: the SNT titre declined more quickly than the cELISA titre, and the kappa value was lower than the overall kappa at the second sampling date (S2) while it improved at the third sampling date (S3) when the ELISA values had increased.

Recently, Oura et al. found that 22/22 lambs fed with maternal colostrum from ewes vaccinated with the Intervet Bovilis BTV-8 inactivated vaccine had protective neutralising antibodies at 10 weeks of age and 14/22 (64%) at 14 weeks of age [22]. The persistence of neutralising antibodies appears slightly longer than in our study (84 days by SNT), possibly due to species differences or study design. Following challenge with BTV-8 at 13 to 14 weeks of age, all lambs were protected from clinical disease and only 5/22 showed transitory BTV RNA. These data show that maternally acquired antibodies directed against BTV can confer long-lasting protection against BTV infection. However, in another study, the duration of protection may well have been shorter, as no neutralising antibodies were found in calves born from BTV-2 vaccinated dams after 39 days of age [23]. It should be noted that several parameters could affect the duration of colostral antibodies, including the type of vaccine, the quantity of antibodies consumed and absorbed from the colostrum, and the rate of decay.

To determine the optimal age for vaccination of calves, it is crucial to know at what time colostral antibodies will have sufficiently waned so as not to interfere with the vaccine. Currently, the recommended age for vaccination of calves with inactivated BTV-8 vaccines varies from 1 to 3 months, depending on the vaccine manufacturer. However, these recommendations have not been based on extensive study, especially on the impact of colostral antibodies on the vaccinal response. Several studies have shown that colostrum-fed or vaccinated animals with neutralising antibodies are generally protected against infection, but that not all animals without neutralising antibodies are fully susceptible [21,22,24]. In the present study, vaccination of colostrum-fed calves with an inactivated viral vaccine poorly induce anti-BTV humoral immunity in most cases (Table 2 and Figure 3). From the data, it appears that antibody levels measured by cELISA are more predictive of vaccinal responsiveness than SNT. Indeed, a few days before vaccination, all strong responders to vaccination displayed cELISA-based PN ≥ 103, while the group of non-responders exhibited mixed positive and negative values, but all with PN < 76. Using SNT, both responders and non-responders showed BTV antibody titres below the detection limit, precluding distinction between the two groups. Moreover, by combining the survival analysis of the BTV colostral antibodies and the responsiveness to vaccination, we could estimate the time required to achieve a vaccination success rate of 50% as falling between 148 and 180 days of age, depending on the cELISA cut-off used; the maximum vaccination success probability would be reached at around 7 months. Due to the small number of animals involved in this survey, these results need to be confirmed and refined in a larger cohort. However, this predictive value confirms the apparent long-lasting presence of a protective humoral immunity against BTV in calves.

It is of critical importance to assure a shift from maternal to vaccination protection without any gap in protection. Even though maternally-derived immunity may block the antibody response in calves, anti-viral vaccination at the earliest possible time may be desirable, as many calves acquire insufficient levels of maternal antibodies [25]. Such calves should therefore respond to vaccination, which raises the issue of the difficulty in designing and implementing a universally successful vaccination program. Since it is not conceivable to measure colostral antibodies before immunization, in order to address the high levels of interference produced from colostral antibodies, it may be wise to propose that: (i) during periods of vector activity (May-October) and potential virus circulation, calves born to vaccinated dams should be vaccinated on two occasions, before three months and then once again around the age of six months in order to ensure maximal protection and provide vaccinal coverage for cases of poor colostrum intake or inefficient vaccination of the dams; (ii) outside these periods, in the absence of BTV circulation, it seems unnecessary to vaccinate before weaning as maternally-derived immunity prevents the humoral response to vaccination, and vaccination around 5-6 months should be adequate. Further investigations on this issue could help to address the revision of current recommendations.

## Competing interests

The authors declare that they have no competing interests.

## Authors' contributions

DV, GB, LB, SZ and GZ drafted the manuscript. JG and FW conceived of the study, and participated in its design and coordination. CS, CV, AD and EB carried out the SNT and cELISA. BD and GZ performed the statistical analysis. All authors read and approved the final manuscript.
